# Vegetable By-Product Lacto-Fermentation as a New Source of Antimicrobial Compounds

**DOI:** 10.3390/microorganisms7120607

**Published:** 2019-11-22

**Authors:** Annalisa Ricci, Valentina Bernini, Antonietta Maoloni, Martina Cirlini, Gianni Galaverna, Erasmo Neviani, Camilla Lazzi

**Affiliations:** Department of Food and Drug, University of Parma, Parco Area delle Scienze 49/A, 43124 Parma, Italy; annalisa.ricci2@studenti.unipr.it (A.R.); valentina.bernini@unipr.it (V.B.); antonietta.maoloni@studenti.unipr.it (A.M.); martina.cirlini@unipr.it (M.C.); gianni.galaverna@unipr.it (G.G.); erasmo.neviani@unipr.it (E.N.)

**Keywords:** antimicrobials, lactic acid fermentation, tomato, carrot, melon, by-products, foodborne pathogens, agar well diffusion assay

## Abstract

Background: One of the main objectives of the food industry is the shelf life extension of food products, taking into account the safety requirements and the preference of consumers attracted by a simple and clear label. Following this direction, many researchers look to find out antimicrobials from natural sources. Methods: Tomato, carrot, and melon by-products were used as substrates for lactic acid fermentation using seven strains belonging to the *Lactobacillus* genus, *L. plantarum, L. casei, L. paracasei*, and *L. rhamnosus*. The obtained fermented by-products were then extracted and the antimicrobial activity toward fourteen pathogenic strains of *Listeria monocytogenes*, *Salmonella spp.*, *Escherichia coli*, *Staphylococcus Aureus*, and *Bacillus cereus* was tested through agar well diffusion assay. Results: All the extracts obtained after fermentation had highlighted antimicrobial activity against each pathogen tested. In particular, a more effective activity was observed against *Salmonella spp., L. monocytogenes, S. aureus*, and *B. cereus*, while a lower activity was observed against *E. coli*. Conclusion: Lactic acid fermentation of vegetable by-products can be a good strategy to obtain antimicrobials useful in food biopreservation.

## 1. Introduction

The increasing occurrence of foodborne illness outbreaks caused by pathogenic microorganisms still represents a threat for consumers [1] and, at the same time, manufacturers are called to manage safety issues in an innovative way. Indeed, chemical additives, commonly used in food products to inhibit microbial growth, improving quality and extending shelf-life, are negatively perceived by consumers [2,3].

In order to ensure food safety while trying to match consumer expectations [2], the research of natural preservatives as an alternative to chemicals represents an interesting avenue [4,5,6]. In this context essential oils, enzymes, peptides, organic acids, chitosan, bacteriocins, and bacteriophages have been considered [7,8].

The antimicrobial effect of some fruits and vegetables, comprising leaves, bulbs, and seeds, has been demonstrated and mainly attributed to the presence of major bioactive compounds such as phenols, terpenes, aliphatic alcohols, aldehydes, acids, and isoflavonoids [4]. Significant antimicrobial activities toward common foodborne pathogens such as *Bacillus cereus* and *Escherichia coli* [9] have also been highlighted after fermentation of vegetable products, opening interesting potential applications for microbial fermentation.

Among microorganisms, many lactic acid bacteria (LAB) have the generally recognized as safe (GRAS) status and can produce antimicrobial compounds such as organic acids (lactic, acetic, or propionic acid), diacetyl, bacteriocins as well as other metabolites. Their formation is strain and species dependent, but it is also related to the characteristics of the fermented substrates. However, nowadays limited information on the use of fermented vegetables as potential antimicrobial agents is available [5]. Vegetable and fruit processing generates a large number of by-products still rich in nutrients and bioactive compounds which may be fermented and metabolized [10].

Among vegetables, tomato (*Lycopersicon esculentum*) represents a symbol of the Mediterranean diet. More than 80% of tomatoes are consumed as processed products [11] which makes the management of their by-products one of the most important sustainability-related issues faced by agro-industrial companies [12]. Tomato by-products contain bioactive compounds such as polyphenols, carotenoids, and vitamins showing a wide range of physiological properties [13]. In the Mediterranean area, other fruit and vegetable productions generate a high quantity of products discarded during harvest because they do not comply with the shapes and sizes required by the processing companies. Among these, melon (*Cucumis melo*) and carrot (*Daucus carota* subsp. *sativus*) are widely cultivated in temperate regions [14] and are characterized by numerous bioactive compounds. Considering the rich composition of all these products and the ability of LAB to produce antimicrobials, the present study was aimed at: (i) screening the aptitude of different LAB strains, belonging to *Lactobacillus plantarum*, *Lactobacillus rhamnosus, Lactobacillus casei*, and *Lactobacillus paracasei* species, to ferment by-products deriving from tomato processing and from carrots and melons discarded during harvest; (ii) in vitro antimicrobial activity evaluation of fermented by-product extracts toward 14 strains of *Salmonella spp.*, *E. coli*, *Staphylococcus aureus*, *Listeria monocytogenes*, and *B. cereus*.

## 2. Results

### 2.1. Tomato, Carrot, and Melon By-Products Lacto-Fermentation

Tomato, carrot, and melon by-products were fermented with seven different LAB strains: *L. plantarum* (285, POM1), *L. casei* (2240, 2246), *L. paracasei* (4186), and *L. rhamnosus* (1019, 1473). The microbial growth was evaluated after 72 h of incubation at the optimal temperature for each species (30 °C for *L. plantarum* and 37 °C for *L. casei*, *L. paracasei*, and *L. rhamnosus*). Overall, all the strains showed a good growth ability with an average value of 1.74 Log CFU g^−1^. Regarding tomato by-products the best growth performance was registered by *L. plantarum* POM1 with an increase of 1.80 Log CFU g^−1^ (Table 1). Only *L. paracasei* 4186 showed an increase lower than 1 Log CFU g^−1^. Instead all the other strains implemented their concentration in a range between 1.01 and 1.62 Log CFU g^−1^. Considering melon by-products, both strains 1019 and 1473, belonging to *L. rhamnosus*, showed the highest increase at the end of fermentation (2.51 and 2.55 Log CFU g^−1^, respectively). On the other hand, *L. paracasei* 4186 highlighted the lowest growth capacity (1.22 Log CFU g^−1^) compared to the other strains (Table 1). Instead, in carrot by-products, a more homogenous behavior was observed (Table 1). 

The pH was measured after fermentation and extraction in all the extracts obtained. It ranged from 4.28 ± 0.04 (non-sterile tomato) to 2.89 ± 1.19 (fermented tomato with *L. casei* 2240) in tomato by-product extracts. As observed for the extracts of tomato by-products, the pH of melon and carrot extracts also decreased after fermentation, moving from 4.57 ± 0.10 (sterile melon) to 3.52 ± 0.04 (fermented melon with *L. casei* 2240) and from 4.44 ± 0.04 (non-sterile carrot) to 3.46 ± 0.01 (fermented carrot with *L. casei* 2240) (Table 2). 

### 2.2. Antimicrobial Activity toward Foodborne Pathogens

**Figure 1:**
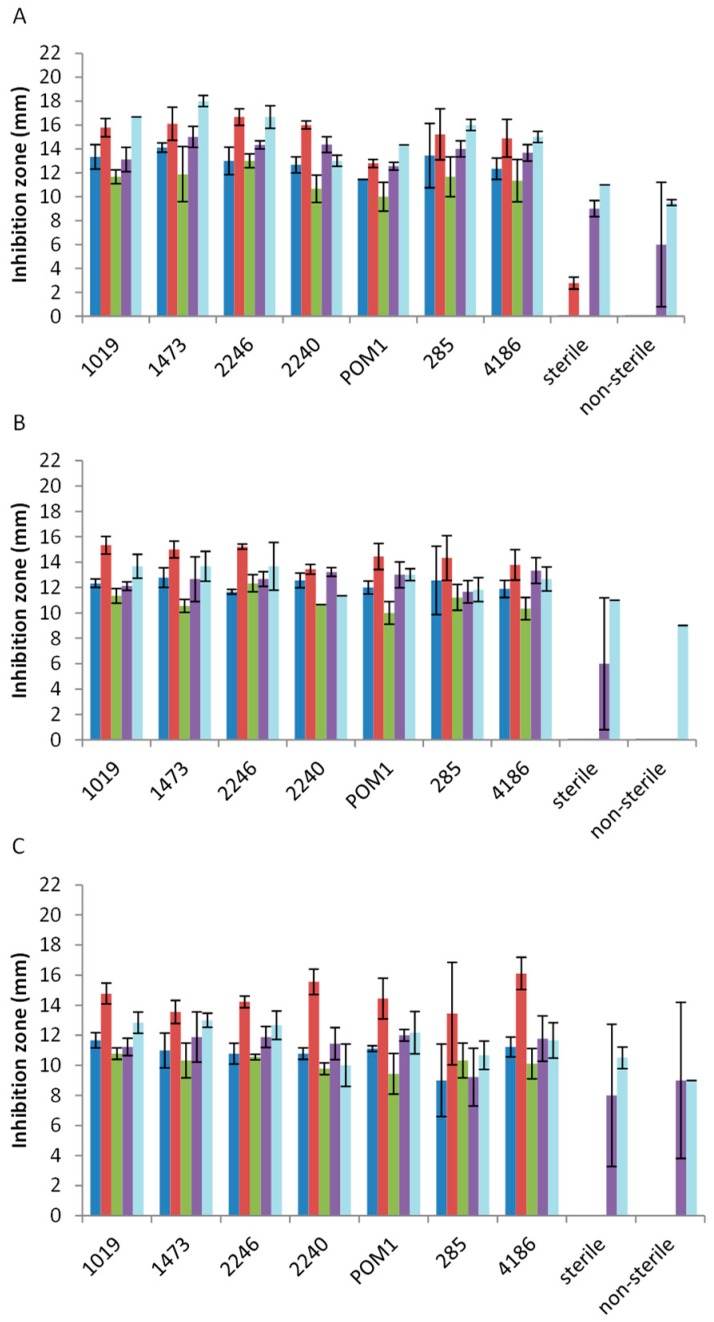
Antimicrobial activity of fermented and not fermented tomato by-product extracts after 24 h (**A**), 48 h (**B**), and 120 h (**C**) of incubation on *Salmonella spp.* (blue line), *Listeria monocytogenes* (red line), *Escherichia coli* (green line), *Staphylococcus aureus* (purple line), and *Bacillus cereus* (light blue line). Size (mm) of inhibition zone is reported.

**Figure 2:**
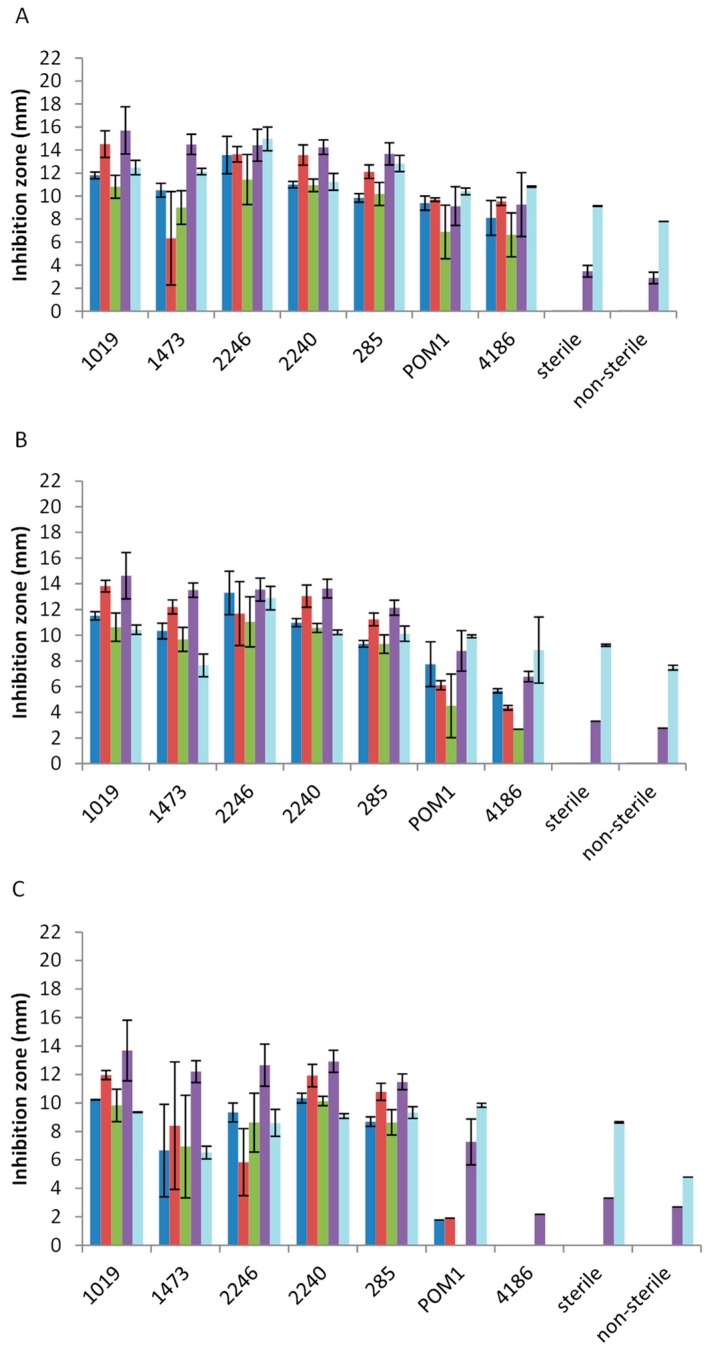
Antimicrobial activity of fermented and not fermented carrot by-product extracts after 24 h (**A**), 48 h (**B**), and 120 h (**C**) of incubation on *Salmonella spp.* (blue line), *L. monocytogenes* (red line), *E. coli* (green line), *S. aureus* (purple line), and *B. cereus* (light blue line). Size (mm) of inhibition zone is reported.

**Figure 3:**
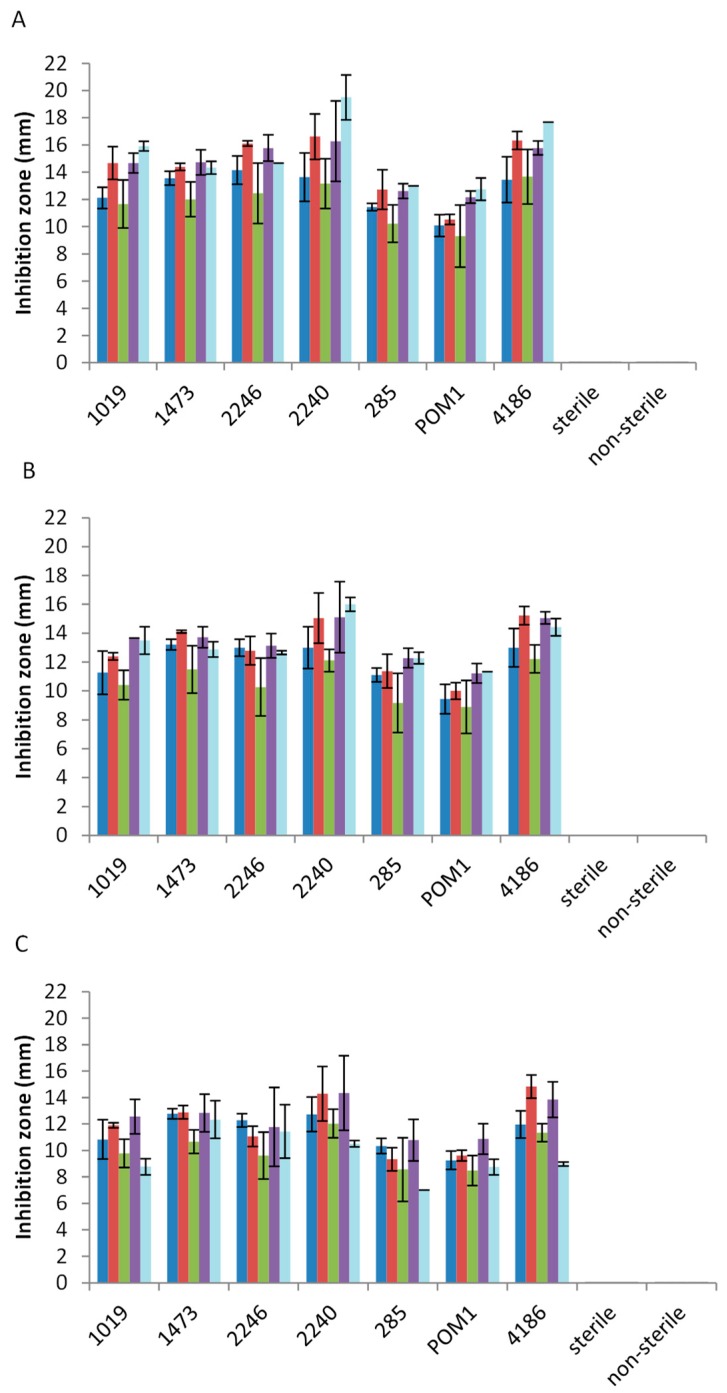
Antimicrobial activity of fermented and not fermented melon by-product extracts after 24 h (**A**), 48 h (**B**), and 120 h (**C**) of incubation on *Salmonella spp.* (blue line), *L. monocytogenes* (red line), *E. coli* (green line), *S. aureus* (purple line), and *B. cereus* (light blue line). Size (mm) of inhibition zone is reported.

The in vitro antimicrobial activity was carried out by agar well diffusion assay which allows the evaluation of the inhibition zone of a microbial cell layer, grown on agar plate, due to a solution spreading in the culture medium. This analysis was carried out using 14 pathogenic strains belonging to *Salmonella spp.*, *L. monocytogenes*, *S. aureus*, *E. coli*, and *B. cereus*. The antimicrobial activities of the extracts were determined by evaluating the presence and size (in mm) of the inhibition zone around the well after 24, 48, and 120 h of plate incubation. The presence of antimicrobial activity in the unfermented by-product extracts, previously sterilized and not, was also verified. To obtain an overview of the extracts’ activity against each pathogenic genus/species tested, the average of the results, reported in Figure 1 (for tomato by-product extracts), Figure 2 (for carrot by-product extracts), and Figure 3 (for melon by-product extracts), are given in the manuscript. The results were comparable among pathogenic genus/species tested and all the extracts’ inhibition zones are reported as Supplementary Material (Tables S1–S3).

Overall, it was observed that the extracts obtained not employing fermentation have lower or no antimicrobial activity compared to those obtained after lacto-fermentation. In particular, in the absence of fermentation, a slight activity was observed only against *B. cereus* and *S. aureus*. In general, the extracts obtained from fermented tomato, carrot, and melon by-products were more effective against *Salmonella spp.*, *L. monocytogenes*, *S. aureus*, and *B. cereus*, while a lower activity was observed toward *E. coli*. 

Tomato by-product extracts exerted anti-*Salmonella* activity after fermentation (Figure 1A), showing a slight decrease after 48 and 120 h (Figure 1B,C), instead no activity was observed for the controls (extracts obtained from sterile and non-sterile tomato). Moreover, *L. monocytogenes* was affected by fermented tomato extracts especially after the employment of strains isolated from dairy products (1019, 1473, 2246, 2240, 285, and 4186) (Figure 1A) maintaining their activity over 120 h (Figure 1B,C). *E. coli* was the species, among those tested, exhibiting the lowest sensibility. Nevertheless, the extracts obtained with *L. rhamnosus* (1019 and 1473) and *L. casei* (2246) showed the highest activity, maintaining it during time (Figure 1A–C). *S. aureus* was inhibited by the fermented extracts, but *B. cereus* was the microorganism most inhibited by tomato and melon extracts especially by *L. casei* group strains after 24 h (Figure 1A). *E. coli* with *S. aureus* and *B. cereus* were the only species inhibited by the controls extracts also, even if only to a lesser extent. 

Moving to carrot extracts, after *L. casei* 2246 fermentation, the highest anti-*Salmonella* activity was observed, while *L. casei* 2246, *L. casei* 2240, and *L. rhamnosus* 1019 showed the strongest inhibition against *L. monocytogenes* (Figure 2A). Overall, for carrot extracts as well, *E. coli* was less sensitive (Figure 2A–C). Regarding *S. aureus,* the extracts obtained after *L. rhamnosus*, *L. casei,* and *L. plantarum* (285) fermentation showed a good antimicrobial activity (Figure 2). However, sterile and non-sterile extracts have also shown antimicrobial activity, but lower compared to the fermented ones. *B. cereus* was inhibited in particular by *L. casei* 2246 fermented extract (Figure 2A) reducing its activity up to 48 and 120 h (Figure 2B,C). As observed for *S. aureus, B. cereus* was also partially inhibited by the control extracts (sterile and non-sterile carrots).

The last extracts tested were derived from melon by-products fermentation. Differently from tomato and carrot by-products, in this case no antimicrobial activity was observed in sterile and non-sterile matrix (Figure 3). In fermented melon by-products, *L. casei* group strains (*L. rhamnosus* 1019 and 1473, *L. casei* 2240 and 2246 and *L. paracasei* 4186) showed the highest anti-*Salmonella* activity (Figure 3A–C). The maximum inhibition values observed for *L. monocytogenes* were highlighted after 24 h employing *L. casei* and *L. paracasei* strains (Figure 3A), remaining stable over time (especially *L. casei* 2240 and *L. paracasei* 4186) (Figure 3B,C). *E. coli* showed the same low sensibility comparable to the other extracts tested in this study. 

Overall, all the fermented extracts showed an antimicrobial activity against the pathogenic microorganisms tested (*L. monocytogenes*, *Salmonella spp.*, *E. coli*, *S. aureus*, and *B. cereus*) independently from the raw matrix, nevertheless tomato and melon exerted the highest activity. 

## 3. Discussion

Nowadays, one of the main goals of food companies is food shelf life extension, in compliance with consumer safety requirements. Following the growing interest of consumers toward products with a simple, clear label and without preservatives perceived as “synthetic”, many researchers aim to find out antimicrobials from natural sources. Several works have focused on the in vitro antimicrobial activity of different plant extracts against the genera *Listeria, Salmonella, Escherichia, Staphylococcus*, and *Bacillus* [15,16,17,18], demonstrating their efficacy. Others reported that fermented products can explain antimicrobial activity [19], even higher than the raw matrix [20] but none of them describe the LAB fermentation as a process applied to vegetable substrates, such as waste and by-products, to obtain antimicrobial extracts.

Fermentation is known to be one of the oldest technologies used by men for various purposes such as the extension of food’s shelf-life, the increase of food safety, and the improvement of nutritional and organoleptic characteristics of final products [21,22,23,24,25]. A LAB feature of industrial interest is their ability to produce antimicrobial metabolites useful in food preservation. As known, LAB can express antimicrobial activity toward pathogenic and alterative microorganisms being able to produce organic acids, thanks to lactic acid fermentation, hydrogen peroxide, CO_2_, and peptides or proteins, such as bacteriocins [26]. Furthermore, some phenolic compounds, showing antimicrobial activity, can be increased or produced ex novo during lactic acid fermentation [27]. Some studies reported the production of phenyllactic acids from LAB and the antimicrobial activity of these compounds has been widely documented on pathogenic microorganisms [28,29,30].

The fermentation process, especially in solid state, can also be used for the low-cost recovery of agro-industrial residues, producing low volumes of wastewaters. To reach these purposes, in this study, the growth of bacteria used as starter for tomato, carrot and melon by-product fermentation was firstly evaluated. The species considered (*L. plantarum, L. rhamnosus, L. casei*, and *L. paracasei*) have shown good growth capacity in these substrates. Although few studies have dealt with the fermentation of these matrices, the data obtained in the present work confirm what was already reported [31,32]. In particular, different LAB species (*L. plantarum, L. casei*, and *Lactobacillus sakei*) grew during carrot fermentation, above all *L. plantarum* which is normally found in plant substrates and whose adaptability in these matrices is well known [33]. Additionally, for melon fermentation, the good replication capacity of *L. plantarum* and *Lactobacillus fermentum* was already reported [34]. Moreover, tomatoes, especially tomato juice, was previously fermented by LAB, showing an excellent growth capacity [35]. 

After lactic acid fermentation with *L. plantarum*, *L. rhamnosus*, *L. casei*, and *L. paracasei* strains an interesting antimicrobial activity, practically absent before fermentation, was observed. This highlighted antimicrobial activity does not seem to be related to the strain’s growth capacity. Indeed, POM1 and 4186 strains, which showed different growth trends in tomato by-products, exhibited similar inhibition zones. The differences in the antimicrobial capacity observed between the extracts does not even seem to be correlated with their pH. The extract obtained from the sterile tomato by-products had an activity, toward *B. cereus*, comparable to the extract obtained after *L. casei* 2240 fermentation even if they had different pH (4.06 ± 0.06 and 2.89 ± 1.19, respectively). Not many studies on fermented plant antimicrobial activity are available, and no one reports the use of lacto-fermented tomato, melon, and carrot by-products. The antimicrobial activity of fermented pomegranate juice and aromatic portulaca plant (*Portulaca oleracea*) has been demonstrated against microorganisms such as *E. coli* and *Bacillus megaterium* [36,37] as well as for various fermented plants, including cloves and green tea, the activity has been observed toward *S. aureus, Pseudomonas aeruginosa,* and *Streptococcus spp*. [38]. Unlike the reported researches, focused on fermentation with *L. plantarum*, in the present work also strains belonging to *L. casei* group (*L. rhamnosus, L. casei*, and *L. paracasei*) were applied, showing, in some cases, better performances. It is known that various LAB species are able to produce antimicrobial compounds including organic acids, CO_2_, H_2_O_2_, alcohols, and bacteriocins [39]. After portulaca fermentation, the authors hypothesized that the antimicrobial activity observed may be due to a synergistic effect between organic acids, fatty acids, and polyphenols but, due to the ability of LAB to produce bacteriocins, their involvement in the antimicrobial activity observed in the fermented vegetables matrices can also be assumed. However phenolic compounds could play a key role as antimicrobial agents, indeed various authors reported their activity toward different pathogenic microorganisms [40,41]. It is noted that different extracts, derived from plant, generally show a greater activity toward gram-positive rather than gram-negative bacteria that could be due to the different structure of the cell wall [16]. The extracts obtained after LAB fermentation, tested in the present work, showed the same trend highlighting for all the tested substrates a greater activity against *L. monocytogenes*, *S. aureus*, and *B. cereus* in comparison to that against *E. coli*. 

## 4. Materials and Methods

### 4.1. Tomato, Carrot, and Melon By-Products

All the by-products ground, sterilized by autoclave at 121 °C for 20 min and not sterilized, were stored at −80 °C until use. 

### 4.2. Lactic Acid Bacteria Strains 

Seven bacterial strains, isolated from both plant and dairy products, belonging to four different lactobacilli, were used for by-product fermentation: *L. plantarum* (POM1 and 285), *L. casei* (2240 and 2246), *L. paracasei* (4186), and *L. rhamnosus* (1019 and 1473). All the strains, with the exception of *L. plantarum* POM1, kindly provided by the Department of Soil, Plants, and Food Sciences (University of Bari, Bari, Italy), belong to the collection of the Department of Food and Drug (University of Parma, Parma, Italy). All the strains were maintained at −80 °C in De Man, Rogosa, and Sharpe (MRS) broth (Oxoid, Basingstoke, UK) supplemented with 12.5% glycerol (v/v).

### 4.3. Setup of Fermentation Conditions 

Before fermentation, LAB strains were transferred twice in MRS broth (Oxoid) (3% v/v) and incubated for 24 h at 30 °C for *L. plantarum* strains and 37 °C for *L. casei, L. paracasei*, and *L. rhamnosus* strains, under aerobic conditions. Afterward, fresh MRS broth was inoculated (3% v/v) with each revitalized strain and incubated for 15 h at the specific temperatures of each species, in order to obtain a cell concentration of 9 Log CFU mL^−1^. Each grown bacterial culture was centrifuged (10,000× *g*, 10 min, 4 °C), washed twice in Ringer solution (Oxoid), and suspended in sterile bidistilled water. The three sterilized substrates (tomato, melon, and carrot by-products) were inoculated individually with each bacterial suspension in order to obtain a final concentration of 7 Log CFU mL^−1^. The inoculated by-products were then incubated for 72 h at 30 °C for *L. plantarum* strains, and at 37 °C for *L. casei, L. paracasei*, and *L. rhamnosus* strains. Bacterial concentration was determined just after inoculum (T0) and at the end of fermentation (72 h). Tenfold dilutions in Ringer solution (Oxoid) were plated in MRS agar (Oxoid), followed by incubation for 48 h in aerobic condition at the specific temperatures for each strain. Twenty-one fermentation conditions, corresponding to the three by-products individually inoculated with seven strains, were carried out in triplicate, for a total of 63 samples that, for each sampling time, were analyzed in duplicate. Average values ± standard deviations were reported.

### 4.4. Extract Production

In order to extract molecules with potential antimicrobial activity such as polyphenols, small peptides, and acids, an extraction process was carried out modifying a protocol previously reported [42]. In particular, water/ethanol 50/50 (v/v) acidified with 0.1% formic acid (CH_2_O_2_) was used as the solvent. Ten grams of lyophilized fermented substrate were homogenized with 100 mL of solvent using an Ultra-Turrax homogenizer (IKA T50 digital, IKA-Werke GmbH & Co., Staufen, Germany) for 5 min at 4000 rpm. The obtained homogenate was then filtered. The liquid part was recovered, while the solid part was subjected to a second extraction using the same condition just described, and eventually the liquid parts obtained from the two extraction were mixed. The extracts obtained were concentrated by the rotary evaporator STRIKE 300 (Steroglass, Perugia, Italy) at 150 rpm with a bath temperature of 40 °C. Then they were resuspended in distilled water in order to obtain solutions concentrated to 60% (w/v) for melon and carrot by-products and to 40% (w/v) for tomato by-products. Non-fermented sterilized and not sterilized tomato, melon, and carrot by-products were used as controls and subjected to the same procedure. The pH of all the extracts was measured in duplicate (Mettler Toledo, Greifensee, Switzerland) and then samples were stored at −80 °C until it was time to screen their antimicrobial activity in vitro conditions. 

### 4.5. Pathogenic Bacterial Strains

The antimicrobial activity of extracts was tested toward 14 pathogenic strains belonging to: *Salmonella spp*. (*S. enterica* ATCC 14028, *S. enterica* serotype Rissen, *Salmonella spp*. suini), *L. monocytogenes* (LM30, LMG 21264, LMG 13305), *E. coli* (DSMZ 9025, DSMZ 10973, POM 1048), *S. aureus* (NCTC 9393, ATCC 6538, ATCC 19095), *B. cereus* (33, 31). The strains used are part of international collections, such as Deustsche Sammlung von Mikroorganismen (DSM), the American Type Culture Collection (ATCC), the Belgian co-ordinated collection of microorganisms (LMG), the National Collection of Type Cultures (NCTC), and the collection of the Department of Food and Drug (University of Parma, Parma, Italy). The strains were kept at −80 °C in tryptic soy broth (TSB) (Oxoid) supplemented with 12.5% glycerol (v/v). Before use, they were cultured twice, for 24 h at 37 °C, with a 3% v/v inoculum in TSB added with 0.6% yeast extract (Oxoid). Each revitalized culture was used to test the sensitivity to the extracts.

### 4.6. Evaluation of In Vitro Antimicrobial Activity 

The antimicrobial activity was carried out employing the agar well diffusion assay [43] with little modifications. The pathogenic strains were diluted until they reached the concentration of 8 Log CFU mL^−1^. A sterile cotton swab was dipped into the obtained suspension and it was seeded on tryptic soy agar (TSA) (Oxoid). Wells with a diameter of 7 mm were created in the agar and filled with the solution to be tested. The antimicrobial activity was evaluated for fermented extracts and controls (sterile and non-sterile by-products) using 30 μL of extract at 60% (for carrot and melon) and 40% (for tomato). Plates were incubated at 37 °C in aerobic conditions and the antimicrobial activity was evaluated by measuring the total inhibition zone diameter (mm) observable after 24, 48, and 120 h of incubation. Analyses were performed in triplicate and average values ± standard deviations were reported.

## 5. Conclusions

To prolong shelf life and maintain the quality of their products, food industries are attempted to find out natural preservatives as an alternative solution to the chemical ones, following the preferences of consumers attracted by simple labels. From the data obtained in this work, it can be highlighted that: (i) Tomato, carrot, and melon by-products are fermentable substrates; (ii) different extracts obtained after fermentation have an antimicrobial activity against different foodborne pathogens such as *L. monocytogenes, Salmonella spp., E. coli, S. aureus*, and *B. cereus;* and (iii) lactic acid fermentation can be a valid method to obtain antimicrobial extracts. These promising results lay the foundation for fermented extracts’ application in food products in order to ensure safety and to extend food shelf life. Future studies will be directed to determine the chemical composition of extracts in order to find out the molecular components responsible for the antimicrobial activity and to highlight their mechanism of action.

## 6. Patents

Patent pending no. 102019000006815.

## Figures and Tables

**Table 1:** Growth ability of different lactic acid bacteria (LAB) species/strains in tomato, melon, and carrot by-products after 72 h of fermentation at the optimal growth temperature (30 °C for *Lactobacillus plantarum* and 37 °C for *Lactobacillus paracasei*, *Lactobacillus casei*, and *Lactobacillus rhamnosus*).

	Tomato			Melon			Carrot		
Strain	T_0_	T_72_	Δ (T_72_ − T_0_)	T_0_	T_72_	Δ (T_72_ − T_0_)	T_0_	T_72_	Δ (T_72_ − T_0_)
*L. plantarum* POM1	7.04 ± 0.10	8.84 ± 0.24	1.80	6.93 ± 0.35	8.88 ± 0.13	1.94	7.26 ± 0.10	9.30 ± 0.08	2.04
*L. plantarum* 285	7.73 ± 0.09	9.35 ± 0.08	1.62	7.62 ± 0.02	9.16 ± 0.08	1.54	7.64 ± 0.05	9.39 ± 0.05	1.75
*L. paracasei* 4186	7.82 ± 0.15	8.66 ± 0.45	0.84	7.86 ± 0.26	9.08 ± 0.21	1.22	7.67 ± 0.07	9.42 ± 0.07	1.75
*L. casei* 2246	7.21 ± 0.29	8.78 ± 0.38	1.57	7.59 ± 0.21	9.48 ± 0.31	1.89	7.60 ± 0.04	9.64 ± 0.06	2.03
*L. casei* 2240	7.68 ± 0.10	9.07 ± 0.09	1.39	7.75 ± 0.07	9.37 ± 0.03	1.61	7.71 ± 0.03	9.68 ± 0.09	1.97
*L. rhamnosus* 1473	7.80 ± 0.09	9.37 ± 0.20	1.57	7.03 ± 0.11	9.57 ± 0.08	2.55	7.43 ± 0.12	9.43 ± 0.11	2.00
*L. rhamnosus* 1019	7.39 ± 0.29	8.40 ± 0.25	1.01	7.36 ± 0.23	9.87 ± 0.56	2.51	7.64 ± 0.05	9.50 ± 0.04	1.86

Data are reported as Log CFU g^−1^ (average values ± standard deviation).

**Table 2:** pH of all the tested extracts. Data are reported as the mean value ± standard deviation.

Extract	Tomato	Melon	Carrot
*L. plantarum* POM1	3.08 ± 0.04	3.70 ± 0.02	3.66 ± 0.02
*L. plantarum* 285	3.70 ± 0.08	3.61 ± 0.02	3.60 ± 0.00
*L. paracasei* 4186	3.19 ± 0.80	3.57 ± 0.02	3.63 ± 0.02
*L. rhamnosus* 1019	3.66 ± 0.03	3.64 ± 0.01	3.47 ± 0.07
*L. rhamnosus* 1473	3.50 ± 0.14	3.62 ± 0.05	3.52 ± 0.05
*L. casei* 2240	2.89 ± 1.19	3.52 ± 0.04	3.46 ± 0.01
*L. casei* 2246	3.51 ± 0.47	3.54 ± 0.06	3.48 ± 0.02
Sterile substrate	4.06 ± 0.06	4.57 ± 0.10	4.33 ± 0.16
Non-sterile substrate	4.28 ± 0.04	4.53 ± 0.13	4.44 ± 0.04

## Supplementary Materials

Supplementary materials can be found at https://www.mdpi.com/2076-2607/7/12/607/s1. Table S1: Inhibition zones, expressed in mm, of all the extract obtained after tomato by-product lacto-fermentation. Table S2. Inhibition zones, expressed in mm, of all the extract obtained after carrot by-product lacto-fermentation. Table S3. Inhibition zones, expressed in mm, of all the extract obtained after melon by-product lacto-fermentation.
