# Supplementary material for: Vegetable By-Product Lacto-Fermentation as a New Source of Antimicrobial Compounds
Source: Microorganisms. 2019 Nov 22;7(12):607. doi: 10.3390/microorganisms7120607 (PMC6956321; doi:10.3390/microorganisms7120607)
Supplement: Supplementary file 1 [file microorganisms-07-00607-s001.pdf]

Table S1. Inhibition zones, expressed in mm, of all the extract obtained after tomato by-products lacto-fermentation. Different LAB were used for the fermentations. *L. plantarum* POM1 and 285, *L. paracasei* 4186, *L. casei* 2246 and 2240 and *L. rhamnosus* 1473 and 1019. The inhibition zones were obtained using 30 µl of extracts concentrated 40 % (w/v).

		Salmonella spp.			*L. monocytogenes*			*E. coli*			*S. aureus*			*B. cereus*	
**Strain**	**Time**	**ATCC14028**	**rissen**	**suini**	**LM30**	**LMG 21264**	**LMG 13305**	**DSM 9025**	**DSM 10973**	**POM 1048**	**NCTC 9393**	**ATCC 6538**	**ATCC 19095**	**31**	**33**
	24 h	13.67 ± 1.15	14.00 ± 1.00	12.33 ± 3.06	16.33 ± 1.15	14.00 ± 3.61	17.00 ± 2.00	13.00 ± 2.00	12.33 ± 2.31	9.67 ± 1.15	12.33 ± 1.15	13.67 ± 2.31	13.33 ± 2.08	16.33 ± 2.31	17.00 ± 2.00
1019	48 h	12.33 ± 1.15	13.00 ± 0.00	11.67 ± 2.31	15.67 ± 2.31	14.00 ± 3.61	16.33 ± 1.15	12.00 ± 1.00	11.67 ± 1.15	10.33 ± 1.15	11.00 ± 0.00	13.00 ± 2.00	12.33 ± 2.08	14.33 ± 1.15	13.00 ± 2.00
	120 h	11.67 ± 1.15	12.33 ± 1.15	11.00 ± 2.00	14.33 ± 1.15	14.00 ± 3.61	16.00 ± 1.15	11.67 ± 1.15	11.00 ± 2.00	9.67 ± 1.15	9.67 ± 1.15	11.33 ± 0.58	12.67 ± 0.58	13.67 ± 1.15	12.00 ± 1.00
	24 h	13.67 ± 1.15	17.00 ± 2.00	11.67 ± 4.16	17.67 ± 2.31	13.67 ± 1.15	17.00 ± 2.00	13.67 ± 1.15	11.67 ± 2.31	10.33 ± 1.15	14.33 ± 1.15	15.67 ± 1.15	15.00 ± 0.00	17.67 ± 1.15	18.33 ± 1.15
1473	48 h	12.33 ± 1.15	15.67 ± 1.15	10.33 ± 3.06	15.67 ± 2.31	13.00 ± 0.00	16.33 ± 1.15	11.67 ± 1.15	10.33 ± 1.15	9.67 ± 1.15	12.00 ± 1.00	13.67 ± 1.15	12.33 ± 1.15	14.33 ± 1.15	13.00 ± 0.00
	120 h	10.33 ± 1.15	13.67 ± 4.16	9.00 ± 3.46	15.00 ± 2.00	9.67 ± 3.06	16.00 ± 1.15	11.67 ± 1.15	9.67 ± 1.15	9.67 ± 1.15	9.67 ± 1.15	13.00 ± 0.00	13.00 ± 0.00	13.67 ± 1.15	12.33 ± 1.15
	24 h	13.00 ± 2.00	12.33 ± 2.31	13.67 ± 3.06	16.33 ± 3.06	16.67 ± 2.52	17.00 ± 2.00	14.33 ± 2.31	12.33 ± 2.31	12.33 ± 3.06	13.67 ± 1.15	14.33 ± 1.15	15.00 ± 2.00	17.00 ± 3.46	16.33 ± 4.16
2246	48 h	12.00 ± 1.00	11.00 ± 3.46	12.00 ± 1.73	15.00 ± 2.00	15.00 ± 2.00	15.67 ± 1.15	12.33 ± 2.31	12.33 ± 1.53	12.33 ± 1.15	12.33 ± 2.31	13.00 ± 2.00	12.67 ± 2.08	13.67 ± 1.15	13.67 ± 3.06
	120 h	11.00 ± 2.00	10.33 ± 2.31	11.00 ± 2.00	13.33 ± 2.08	14.33 ± 1.15	15.00 ± 2.00	11.00 ± 2.00	10.33 ± 1.15	10.33 ± 2.31	10.67 ± 0.58	12.33 ± 1.15	12.67 ± 2.08	13.67 ± 1.15	11.67 ± 1.15
	24 h	12.67 ± 2.08	12.67 ± 2.08	12.67 ± 0.58	16.33 ± 3.06	15.67 ± 2.08	16.00 ± 2.65	12.00 ± 1.00	9.67 ± 3.06	10.33 ± 2.31	14.00 ± 1.73	14.67 ± 2.52	14.40 ± 1.97	13.00 ± 3.46	13.00 ± 3.46
2240	48 h	12.67 ± 2.08	13.00 ± 2.00	12.00 ± 1.00	12.33 ± 2.31	14.33 ± 2.31	13.67 ± 1.15	11.67 ± 1.15	10.33 ± 2.31	10.00 ± 1.73	12.33 ± 1.15	13.00 ± 2.00	14.33 ± 3.06	11.67 ± 1.15	11.00 ± 2.00
	120 h	10.67 ± 0.58	10.67 ± 0.58	11.00 ± 0.00	14.33 ± 2.31	15.33 ± 1.53	17.00 ± 1.00	11.33 ± 1.53	9.00 ± 2.00	9.00 ± 0.00	11.00 ± 0.00	11.67 ± 0.58	11.67 ± 1.15	11.00 ± 0.00	9.00 ± 0.00
	24 h	13.00 ± 4.00	13.67 ± 3.06	13.67 ± 1.15	16.33 ± 1.15	15.67 ± 1.15	13.67 ± 1.15	13.00 ± 3.46	13.00 ± 0.00	9.00 ± 0.00	13.33 ± 1.53	13.67 ± 2.31	15.00 ± 0.00	16.33 ± 1.15	15.67 ± 1.15
285	48 h	11.67 ± 3.06	13.00 ± 2.00	13.00 ± 2.00	14.33 ± 1.15	15.00 ± 0.00	13.67 ± 1.15	11.67 ± 1.15	11.33 ± 0.58	10.67 ± 1.53	10.33 ± 1.15	11.00 ± 2.00	13.67 ± 1.15	12.67 ± 0.58	11.00 ± 0.00
	120 h	8.33 ± 2.31	10.33 ± 2.31	8.33 ± 2.31	14.33 ± 1.15	13.00 ± 2.00	13.00 ±	11.00 ± 2.00	11.00 ± 0.00	9.00 ± 0.00	7.67 ± 1.15	9.00 ± 2.00	11.00 ± 0.00	11.00 ± 0.00	10.33 ± 1.15
	24 h	11.67 ± 1.15	12.33 ± 1.15	10.33 ± 0.58	12.33 ± 2.31	12.33 ± 2.31	13.67 ± 1.15	10.67 ± 0.58	9.67 ± 1.15	9.67 ± 1.15	11.67 ± 3.06	12.33 ± 2.31	13.67 ± 2.08	14.33 ± 2.31	14.33 ± 2.31
POM1	48 h	11.67 ± 1.15	12.33 ± 1.15	12.00 ± 1.73	15.00 ± 0.00	14.67 ± 2.08	13.67 ± 1.15	10.67 ± 0.58	9.67 ± 1.15	9.67 ± 1.15	12.67 ± 2.08	13.00 ± 2.00	13.33 ± 1.53	13.67 ± 1.15	12.33 ± 1.15
	120 h	10.67 ± 0.58	11.67 ± 1.15	11.00 ± 1.00	15.00 ± 2.00	13.67 ± 1.15	14.67 ± 2.08	9.67 ± 0.58	9.67 ± 1.15	9.00 ± 2.00	11.33 ± 0.58	12.33 ± 1.15	12.33 ± 1.15	12.67 ± 2.08	11.67 ± 1.15
	24 h	11.67 ± 1.15	11.67 ± 1.53	13.67 ± 1.15	14.33 ± 1.15	15.67 ± 4.62	14.67 ± 2.08	12.00 ± 1.73	11.00 ± 2.00	11.00 ± 2.00	13.67 ± 1.15	13.33 ± 1.53	14.00 ± 1.00	15.67 ± 2.08	14.33 ± 2.31
4186	48 h	12.00 ± 1.73	11.67 ± 1.53	12.00 ± 1.73	13.67 ± 1.15	13.67 ± 3.06	14.00 ± 1.00	11.00 ± 2.00	10.33 ± 1.15	9.67 ± 1.15	13.00 ± 0.00	13.00 ± 2.00	14.00 ± 1.00	14.00 ± 1.00	11.33 ± 0.58
	120 h	11.00 ± 2.00	10.67 ± 2.08	12.00 ± 2.65	15.67 ± 1.15	16.33 ± 4.16	16.33 ± 2.31	10.33 ± 2.31	10.00 ± 1.00	10.00 ± 1.73	11.00 ± 0.00	12.00 ± 1.73	12.33 ± 1.15	12.33 ± 1.15	11.00 ± 0.00
	24 h	ND	ND	ND	ND	8.33 ± 1.15	ND	ND	ND	ND	8.33 ± 1.15	9.67 ± 1.15	9.00 ± 0.00	11.00 ± 0.00	11.00 ± 0.00
sterile	48 h	ND	ND	ND	ND	ND	ND	ND	ND	ND	ND	9.00 ± 0.00	9.00 ± 0.00	11.00 ± 0.00	11.00 ± 0.00
	120 h	ND	ND	ND	ND	ND	ND	ND	ND	ND	ND	9.00 ± 0.00	7.00 ± 0.00	11.00 ± 0.00	10.00 ± 0.00
	24 h	ND	ND	ND	ND	ND	ND	ND	ND	ND	9.00 ± 0.00	9.00 ± 0.00	ND	9.33 ± 0.58	9.67 ± 1.15
non-sterile	48 h	ND	ND	ND	ND	ND	ND	ND	ND	ND	ND	ND	ND	9.00 ± 0.00	9.00 ± 0.00
	120 h	ND	ND	ND	ND	ND	ND	ND	ND	ND	ND	9.00 ± 0.00	9.00 ± 0.00	9.00 ± 0.00	9.00 ± 0.00

ND: not detected

Table S2. Inhibition zones, expressed in mm, of all the extract obtained after carrot by-products lacto-fermentation. Different LAB were used for the fermentations. *L. plantarum* POM1 and 285, *L. paracasei* 4186, *L. casei* 2246 and 2240 and *L. rhamnosus* 1473 and 1019. The inhibition zones were obtained using 30 µl of extracts concentrated 60 % (w/v).

		Salmonella spp.			*L. monocytogenes*			*E. coli*			*S. aureus*			*B. cereus*	
**Strain**	**Time**	**ATCC14028**	**rissen**	**suini**	**LM30**	**LMG 21264**	**LMG 13305**	**DSM 9025**	**DSM 10973**	**POM 1048**	**NCTC 9393**	**ATCC 6538**	**ATCC 19095**	**31**	**33**
	24 h	12.07 ± 1.68	11.83 ± 1.44	11.50 ± 0.87	15.83 ± 1.44	13.73 ± 1.03	13.95 ± 1.23	11.49 ± 2.05	11.27 ± 3.44	9.67 ± 1.15	14.17 ± 1.44	14.91 ± 0.63	18.03 ± 1.34	12.03 ± 2.94	12.91 ± 2.76
1019	48 h	11.83 ± 1.89	11.50 ± 1.32	11.20 ± 0.72	14.33 ± 1.15	13.47 ± 0.81	13.67 ± 1.53	11.50 ± 1.80	11.00 ± 3.46	9.40 ± 1.06	13.45 ± 1.18	13.73 ± 0.64	16.70 ± 1.21	10.17 ± 1.24	10.68 ± 2.03
	120 h	10.23 ± 0.75	10.21 ± 0.89	10.24 ± 0.99	12.29 ± 1.12	11.64 ± 2.29	11.95 ± 1.07	10.52 ± 2.60	10.45 ± 3.94	8.51 ± 0.28	11.66 ± 1.38	13.45 ± 0.51	15.91 ± 0.43	9.33 ± 1.89	9.37 ± 2.83
	24 h	11.00 ± 0.00	10.67 ± 0.58	9.83 ± 1.04	3.67 ± 6.35	4.33 ± 7.51	11.00 ± 3.46	7.33 ± 6.35	9.67 ± 1.15	10.00 ± 1.00	13.47 ± 0.50	15.00 ± 0.00	15.00 ± 0.00	12.33 ± 1.15	11.93 ± 1.36
1473	48 h	10.87 ± 0.23	10.45 ± 0.64	9.67 ± 1.15	12.75 ± 2.05	12.20 ± 1.06	11.67 ± 1.15	10.73 ± 0.23	9.25 ± 0.43	9.03 ± 0.45	12.88 ± 0.55	13.75 ± 0.25	13.91 ± 1.01	7.03 ± 6.09	8.28 ± 7.17
	120 h	8.57 ± 0.19	8.49 ± 0.45	2.91 ± 5.05	3.25 ± 5.62	11.29 ± 0.84	10.67 ± 0.29	9.58 ± 1.00	2.83 ± 4.91	8.41 ± 0.48	11.33 ± 0.41	12.57 ± 0.26	12.71 ± 0.61	6.20 ± 5.56	6.83 ± 5.97
	24 h	15.41 ± 0.38	12.87 ± 4.20	12.40 ± 3.08	14.40 ± 1.22	13.17 ± 4.65	13.33 ± 3.21	13.73 ± 2.19	9.41 ± 2.45	11.13 ± 2.42	12.83 ± 0.52	15.20 ± 3.04	15.25 ± 1.15	14.25 ± 1.74	15.70 ± 1.92
2246	48 h	15.17 ± 0.76	12.80 ± 4.30	11.91 ± 2.60	13.40 ± 1.06	8.83 ± 7.65	12.83 ± 3.18	13.08 ± 2.27	9.20 ± 2.60	10.83 ± 2.57	12.73 ± 0.46	13.41 ± 2.50	14.50 ± 1.32	13.53 ± 0.96	12.24 ± 0.50
	120 h	9.33 ± 8.14	10.00 ± 8.89	8.67 ± 7.57	ND	8.33 ± 7.23	11.67 ± 3.06	8.67 ± 7.77	6.53 ± 6.15	10.67 ± 2.52	11.03 ± 0.45	12.98 ± 2.81	13.93 ± 1.29	9.27 ± 8.06	7.93 ± 6.88
	24 h	11.28 ± 0.50	10.71 ± 0.61	11.00 ± 0.35	14.41 ± 1.70	12.67 ± 1.44	13.62 ± 0.21	11.33 ± 0.76	10.33 ± 0.36	11.17 ± 0.29	14.00 ± 1.00	14.97 ± 0.55	13.77 ± 0.25	11.75 ± 0.66	10.73 ± 0.52
2240	48 h	11.33 ± 0.75	10.74 ± 0.78	10.87 ± 0.33	13.94 ± 1.51	12.24 ± 1.18	12.95 ± 0.83	10.96 ± 0.70	10.29 ± 0.59	10.45 ± 0.91	12.91 ± 0.43	14.36 ± 0.50	13.65 ± 0.47	10.10 ± 0.37	10.34 ± 0.97
	120 h	10.70 ± 0.73	10.29 ± 1.08	10.01 ± 0.37	12.61 ± 1.96	11.08 ± 0.88	12.07 ± 0.50	10.49 ± 1.09	9.87 ± 0.37	10.03 ± 1.16	12.12 ± 0.34	13.65 ± 0.26	12.99 ± 0.58	9.20 ± 0.39	8.96 ± 0.81
	24 h	9.66 ± 0.39	9.58 ± 1.00	10.27 ± 0.31	11.58 ± 0.62	12.00 ± 1.32	12.75 ± 0.66	11.17 ± 0.29	9.17 ± 0.29	10.21 ± 0.26	12.58 ± 0.39	14.41 ± 0.95	14.00 ± 0.50	13.33 ± 1.53	12.33 ± 0.58
285	48 h	9.41 ± 0.52	9.04 ± 1.28	9.53 ± 0.73	10.66 ± 1.04	11.45 ± 1.43	11.57 ± 0.75	10.12 ± 0.34	8.75 ± 0.44	9.08 ± 0.39	11.53 ± 0.29	12.68 ± 0.35	12.20 ± 0.64	10.53 ± 0.64	9.70 ± 0.18
	120 h	8.99 ± 0.45	8.33 ± 0.69	8.74 ± 0.38	10.12 ± 0.34	11.31 ± 1.04	10.91 ± 0.19	9.66 ± 0.07	8.11 ± 0.22	8.12 ± 0.57	11.20 ± 0.32	12.12 ± 0.69	11.12 ± 1.02	9.61 ± 1.56	9.03 ± 0.26
	24 h	9.87 ± 0.87	8.70 ± 0.94	9.62 ± 0.76	9.54 ± 0.56	9.71 ± 0.83	9.82 ± 0.50	8.53 ± 0.41	ND	5.25 ± 4.55	7.29 ± 6.56	10.56 ± 1.20	9.53 ± 1.61	10.21 ± 0.71	10.61 ± 0.90
POM1	48 h	8.83 ± 0.51	5.75 ± 4.99	8.67 ± 0.58	ND	8.91 ± 0.80	9.41 ± 0.88	8.50 ± 0.50	ND	5.00 ± 4.33	6.99 ± 6.21	9.99 ± 1.20	9.37 ± 1.56	9.99 ± 0.95	9.83 ± 0.19
	120 h	ND	ND	5.33 ± 4.66	ND	ND	5.73 ± 4.97	ND	ND	ND	6.33 ± 5.51	6.33 ± 5.51	9.11 ± 1.57	9.93 ± 1.01	9.73 ± 0.12
	24 h	6.39 ± 5.71	9.13 ± 0.75	8.79 ± 1.14	9.17 ± 1.76	9.84 ± 1.89	9.61 ± 1.24	8.83 ± 0.76	5.74 ± 4.98	5.33 ± 4.62	6.08 ± 5.44	10.58 ± 0.73	11.12 ± 0.34	10.79 ± 0.37	10.87 ± 0.13
4186	48 h	ND	8.61 ± 0.38	8.38 ± 0.72	6.67 ± 5.86	ND	6.41 ± 5.67	8.03 ± 0.73	ND	ND	ND	9.89 ± 1.02	10.45 ± 0.57	10.67 ± 0.38	7.03 ± 6.09
	120 h	ND	ND	ND	ND	ND	ND	ND	ND	ND	ND	6.53 ± 5.71	ND	ND	ND
	24 h	ND	ND	ND	ND	ND	ND	ND	ND	ND	ND	10.41 ± 0.63	ND	9.11 ± 0.58	9.17 ± 0.29
sterile	48 h	ND	ND	ND	ND	ND	ND	ND	ND	ND	ND	9.93 ± 0.83	ND	9.28 ± 0.60	9.17 ± 0.29
	120 h	ND	ND	ND	ND	ND	ND	ND	ND	ND	ND	9.93 ± 0.83	ND	8.60 ± 1.00	8.67 ± 0.58
	24 h	ND	ND	ND	ND	ND	ND	ND	ND	ND	ND	8.67 ± 0.58	ND	7.80 ± 0.35	7.80 ± 0.35
non-sterile	48 h	ND	ND	ND	ND	ND	ND	ND	ND	ND	ND	8.33 ± 0.31	ND	7.60 ± 0.53	7.33 ± 0.12
	120 h	ND	ND	ND	ND	ND	ND	ND	ND	ND	ND	8.07 ± 0.50	ND	4.80 ± 4.16	4.80 ± 4.16

ND: not detected

Table S3. Inhibition zones, expressed in mm, of all the extract obtained after melon by-products lacto-fermentation. Different LAB were used for the fermentations. *L. plantarum* POM1 and 285, *L. paracasei* 4186, *L. casei* 2246 and 2240 and *L. rhamnosus* 1473 and 1019. The inhibition zones were obtained using 30 µl of extracts concentrated 60 % (w/v).

Strain	Time	*Salmonella spp.*			*L. monocytogenes*			*E. coli*			*S. aureus*			*B. cereus*	
		ATCC14028	rissen	suini	LM30	LMG 21264	LMG 13305	DSM 9025	10973	POM 1048	NCTC 9393	ATCC 6538	ATCC 19095	31	33
1019	24 h	11.67 ± 3.06	13.00 ± 2.00	11.67 ± 2.31	16.00 ± 3.61	14.33 ± 1.15	13.67 ± 1.15	13.67 ± 1.15	10.33 ± 1.15	11.00 ± 0.00	14.33 ± 1.15	14.17 ± 2.02	15.50 ± 0.87	16.17 ± 1.04	15.67 ± 1.15
	48 h	10.50 ± 2.29	13.00 ± 2.00	10.33 ± 3.06	12.33 ± 2.31	12.17 ± 1.04	12.67 ± 2.08	11.58 ± 1.00	9.67 ± 1.15	10.00 ± 1.00	13.67 ± 1.15	13.67 ± 1.15	13.67 ± 1.15	14.17 ± 1.04	12.83 ± 1.76
	120 h	10.33 ± 2.08	12.50 ± 1.32	9.67 ± 2.36	12.00 ± 2.65	11.67 ± 0.76	12.00 ± 1.11	11.00 ± 0.00	9.33 ± 1.53	9.00 ± 1.00	11.07 ± 1.68	13.45 ± 1.32	13.17 ± 0.76	9.20 ± 0.72	8.33 ± 0.58
1473	24 h	13.67 ± 1.15	13.00 ± 0.00	14.00 ± 0.00	14.67 ± 0.58	14.33 ± 1.15	14.17 ± 1.44	13.43 ± 0.40	11.00 ± 1.73	11.57 ± 1.21	13.83 ± 1.04	15.67 ± 1.15	14.67 ± 0.58	14.00 ± 1.73	14.67 ± 2.52
	48 h	13.57 ± 1.25	12.83 ± 0.29	13.25 ± 0.91	14.00 ± 0.00	14.17 ± 1.04	14.17 ± 1.04	13.33 ± 0.29	11.00 ± 0.00	10.17 ± 0.76	13.08 ± 0.88	14.50 ± 0.50	13.58 ± 0.52	13.25 ± 1.09	12.50 ± 0.50
	120 h	13.00 ± 0.00	13.00 ± 2.00	12.33 ± 1.15	13.33 ± 0.58	13.00 ± 2.00	12.33 ± 1.15	11.67 ± 1.15	10.33 ± 1.15	10.00 ± 1.00	11.33 ± 0.58	14.17 ± 1.04	13.00 ± 1.00	13.33 ± 1.15	11.33 ± 2.08
2246	24 h	15.00 ± 0.00	13.00 ± 0.00	14.47 ± 0.50	16.00 ± 1.00	16.00 ± 1.00	16.33 ± 1.15	15.00 ± 0.00	11.33 ± 0.58	11.00 ± 0.00	14.67 ± 1.15	16.33 ± 1.15	16.33 ± 1.15	14.67 ± 0.58	14.67 ± 0.58
	48 h	13.41 ± 0.72	12.33 ± 0.76	13.25 ± 0.66	11.87 ± 1.03	12.67 ± 1.04	13.83 ± 1.04	12.17 ± 0.76	10.48 ± 0.45	8.17 ± 1.26	12.17 ± 1.15	13.67 ± 1.26	13.58 ± 1.38	12.75 ± 0.44	12.57 ± 1.01
	120 h	12.75 ± 0.44	11.75 ± 1.09	12.33 ± 1.15	11.87 ± 1.03	11.00 ± 3.46	10.33 ± 3.06	11.17 ± 0.29	10.00 ± 1.00	7.67 ± 1.15	8.33 ± 0.58	13.41 ± 1.43	13.58 ± 1.67	10.00 ± 2.65	12.87 ± 0.23
2240	24 h	12.92 ± 2.56	12.33 ± 3.06	15.67 ± 1.53	16.00 ± 2.65	18.50 ± 2.78	15.33 ± 1.44	15.25 ± 2.23	12.41 ± 3.45	11.83 ± 1.04	13.00 ± 1.73	17.08 ± 3.51	18.75 ± 1.14	18.33 ± 2.08	20.67 ± 3.79
	48 h	12.33 ± 2.02	12.00 ± 2.65	14.67 ± 2.08	14.50 ± 2.18	17.00 ± 2.00	13.67 ± 1.15	13.00 ± 2.00	11.67 ± 4.16	11.67 ± 1.15	12.33 ± 1.44	16.00 ± 2.65	17.00 ± 2.00	15.67 ± 2.31	16.33 ± 2.08
	120 h	12.03 ± 2.12	11.91 ± 2.55	14.25 ± 1.64	13.16 ± 1.37	16.67 ± 1.76	13.03 ± 1.45	13.25 ± 1.56	11.67 ± 3.51	11.19 ± 0.91	11.08 ± 1.38	15.75 ± 2.70	16.17 ± 1.04	10.67 ± 1.61	10.33 ± 4.04
285	24 h	11.13 ± 0.81	11.60 ± 0.53	11.60 ± 0.53	11.50 ± 0.50	12.33 ± 1.53	14.33 ± 1.15	11.33 ± 0.58	8.67 ± 0.58	10.67 ± 3.79	13.00 ± 0.00	12.83 ± 1.26	12.00 ± 1.73	13.00 ± 0.00	13.00 ± 0.00
	48 h	10.83 ± 0.29	10.83 ± 0.29	11.67 ± 0.58	10.83 ± 0.76	10.57 ± 0.51	12.73 ± 2.05	11.50 ± 0.87	8.33 ± 1.15	7.67 ± 1.15	13.00 ± 0.00	12.17 ± 1.04	11.67 ± 1.53	12.57 ± 0.51	12.00 ± 1.00
	120 h	10.67 ± 0.58	10.67 ± 0.58	9.67 ± 1.15	9.67 ± 1.15	8.33 ± 1.15	10.00 ± 1.73	11.33 ± 0.58	7.33 ± 0.58	7.00 ± 0.00	9.00 ± 0.00	12.00 ± 1.00	11.33 ± 1.15	7.00 ± 0.00	7.00 ± 0.00
POM1	24 h	10.83 ± 0.29	9.25 ± 2.16	10.17 ± 1.89	10.17 ± 1.04	10.91 ± 0.80	10.50 ± 1.32	11.91 ± 1.13	8.33 ± 1.15	7.67 ± 0.58	11.67 ± 1.89	12.50 ± 0.50	12.33 ± 2.08	12.17 ± 1.26	13.33 ± 1.15
	48 h	10.33 ± 1.15	8.33 ± 1.53	9.67 ± 2.31	9.67 ± 1.15	10.67 ± 1.53	9.67 ± 2.31	11.00 ± 0.00	8.00 ± 1.00	7.67 ± 0.58	10.50 ± 0.87	11.83 ± 0.29	11.33 ± 1.15	11.33 ± 0.58	11.33 ± 1.15
	120 h	9.87 ± 1.21	8.50 ± 1.32	9.40 ± 1.59	9.33 ± 1.53	10.07 ± 1.01	9.41 ± 1.23	9.78 ± 0.72	8.00 ± 1.00	7.67 ± 0.58	9.53 ± 1.29	11.66 ± 0.62	11.41 ± 0.80	8.33 ± 0.58	9.17 ± 2.93
4186	24 h	15.00 ± 0.00	13.67 ± 1.15	11.67 ± 1.15	15.67 ± 1.15	17.00 ± 0.00	16.33 ± 1.15	15.67 ± 1.15	11.67 ± 0.58	13.67 ± 0.58	15.33 ± 0.58	15.67 ± 1.15	16.33 ± 1.15	17.67 ± 1.15	17.67 ± 1.15
	48 h	14.33 ± 1.15	13.00 ± 1.00	11.67 ± 1.15	14.50 ± 2.18	15.67 ± 1.15	15.50 ± 1.32	13.33 ± 0.58	11.58 ± 0.52	11.75 ± 0.66	15.00 ± 0.00	15.50 ± 1.32	14.67 ± 0.58	14.00 ± 1.00	14.83 ± 1.61
	120 h	12.81 ± 0.51	12.23 ± 1.17	10.83 ± 1.76	13.83 ± 1.04	15.50 ± 1.32	15.15 ± 1.27	12.07 ± 0.92	10.70 ± 1.08	11.25 ± 0.43	12.28 ± 0.60	14.70 ± 0.82	14.53 ± 0.50	9.07 ± 1.85	8.87 ± 1.50
Sterile	24 h	ND	ND	ND	ND	ND	ND	ND	ND	ND	ND	ND	ND	ND	ND
	48 h	ND	ND	ND	ND	ND	ND	ND	ND	ND	ND	ND	ND	ND	ND
	120 h	ND	ND	ND	ND	ND	ND	ND	ND	ND	ND	ND	ND	ND	ND
non-sterile	24 h	ND	ND	ND	ND	ND	ND	ND	ND	ND	ND	ND	ND	ND	ND
	48 h	ND	ND	ND	ND	ND	ND	ND	ND	ND	ND	ND	ND	ND	ND
	120 h	ND	ND	ND	ND	ND	ND	ND	ND	ND	ND	ND	ND	ND	ND

ND: not detected
